# Biological and Biochemical Characterization of Coronado Island Rattlesnake (*Crotalus helleri caliginis*) Venom and Antivenom Neutralization

**DOI:** 10.3390/toxins13080582

**Published:** 2021-08-21

**Authors:** Cristian Franco-Servín, Edgar Neri-Castro, Melisa Bénard-Valle, Alejandro Alagón, Ramsés Alejandro Rosales-García, Raquel Guerrero-Alba, José Emanuel Poblano-Sánchez, Marcelo Silva-Briano, Alma Lilián Guerrero-Barrera, José Jesús Sigala-Rodríguez

**Affiliations:** 1 Laboratorio de Biología Celular y Tisular, Departamento de Morfología, Centro de Ciencias Básicas, Universidad Autónoma de Aguascalientes, Av. Universidad 940, Aguascalientes CP 20131, Ags, Mexico; cristian@extherapeutics.com; 2 Colección Zoológica, Departamento de Biología, Centro de Ciencias Básicas, Universidad Autónoma de Aguascalientes, Av. Universidad 940, Aguascalientes CP 20131, Ags, Mexico; ramsesr@g.clemson.edu; 3 Instituto de Biotecnología, Universidad Nacional Autónoma de México, Av. Universidad # 2001 Colonia Chamilpa, Cuernavaca CP 62210, Morelos, Mexico; edgar.neri@ibt.unam.mx (E.N.-C.); mel@ibt.unam.mx (M.B.-V.); alejandro.alagon@ibt.unam.mx (A.A.); 4 Laboratorio de Electrofisiología, Departamento de Fisiología y Farmacología, Centro de Ciencias Básicas, Universidad Autónoma de Aguascalientes, Av. Universidad 940, Aguascalientes CP 20131, Ags, Mexico; raquel.guerrero@edu.uaa.mx; 5 Laboratorio Clínico de Especialidades del Hospital General ISSSTE, Av. Universidad 410, Aguascalientes CP 20010, Ags, Mexico; emanuel_poblano@hotmail.com; 6 Laboratorio de Ecología, Departamento de Biología, Centro de Ciencias Básicas, Universidad Autónoma de Aguascalientes, Av. Universidad 940, Aguascalientes CP 20131, Ags, Mexico; msilva@correo.uaa.mx

**Keywords:** island rattlesnake, *Crotalus helleri caliginis*, crotamine isoforms, muscle paralysis, commercial Mexican antivenoms

## Abstract

The Baja California Peninsula has over 250 islands and islets with many endemic species. Among them, rattlesnakes are the most numerous but also one of the least studied groups. The study of island rattlesnake venom could guide us to a better understanding of evolutionary processes and the description of novel toxins. *Crotalus helleri caliginis* venom samples were analyzed to determine possible ontogenetic variation with SDS-PAGE in one and two dimensions and with RP-HPLC. Western Blot, ELISA, and amino-terminal sequencing were used to determine the main components of the venom. The biological and biochemical activities demonstrate the similarity of *C. helleri caliginis* venom to the continental species *C. helleri helleri*, with both having low proteolytic and phospholipase A_2_ (PLA_2_) activity but differing due to the absence of neurotoxin (crotoxin-like) in the insular species. The main components of the snake venom were metalloproteases, serine proteases, and crotamine, which was the most abundant toxin group (30–35% of full venom). The crotamine was isolated using size-exclusion chromatography where its functional effects were tested on mouse phrenic nerve–hemidiaphragm preparations in which a significant reduction in muscle twitch contractions were observed. The two Mexican antivenoms could neutralize the lethality of *C. helleri caliginis* venom but not the crotamine effects.

## 1. Introduction

There are currently more than 3800 species of snakes distributed around the world; however, only three families represent a risk to human health, Atractaspididae with 69 species, Elapidae with 377, and Viperidae with 361 [1,2,3]. Insular distribution is common in Elapidae and Viperidae with 57 and 51 insular species, respectively. Fifteen snake species live on Mexican islands in the Baja California Peninsula, including the Sea of Cortes. *Crotalus* is the most frequently found genus of insular vipers [1,4,5].

The Baja California Peninsula (BCP) is the second longest peninsula in the world, having 250 islands and islets in the Pacific Ocean and the Sea of Cortes [6], and containing many endemic species of mammals, birds, amphibians, and reptiles. There are two snakes of the Elapidae family and 15 species of rattlesnakes (Viperidae family) on the BCP and on 29 of the 250 islands, seven of which are endemic to their respective islands [4,7].

**Figure 1:**
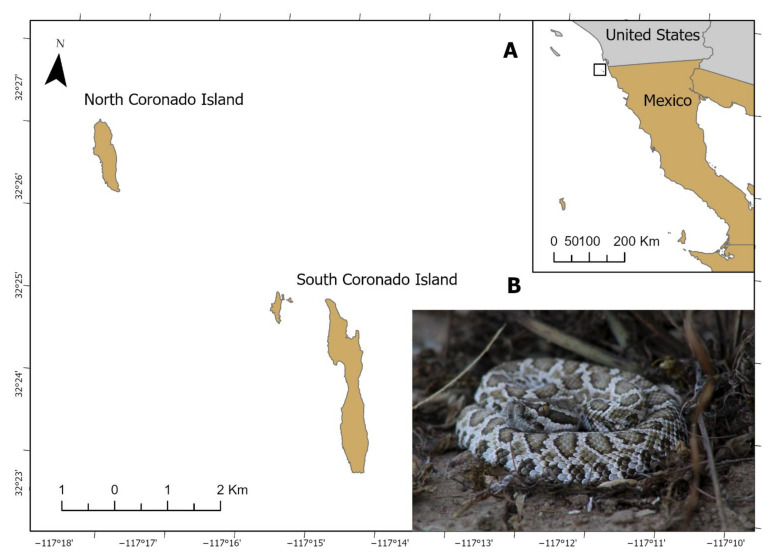
South Coronado Island, Baja California, Mexico (**A**). Juvenile *C. helleri caliginis* (**B**).

The Coronado Islands are a group of three islands (North, Middle, and South Island) and one islet (Middle Rock), located approximately 16 km northwest of Rosarito Beach, Baja California (Mexico) and 26 km southwest of San Diego, CA (USA) [8]. South Coronado Island (Figure 1A) is the largest at nearly 3 km long, 800 m across at its widest part, and 200 m at its narrowest point. Fossil-based studies suggest a Middle Miocene origin for the Coronado Islands [8].

Eight species of reptiles inhabit South Coronado Island: five lizards and three snakes, including South Coronado Rattlesnake, *Crotalus helleri caliginis* (Figure 1B), which is a medium-sized snake, with a 800 mm snout–vent length in the longest specimen, that feeds on lizards and birds, and rarely on the only available mouse species, *Peromyscus maniculatus assimilis*, that is also endemic to the island [9]. The island was likely colonized by this rattlesnake during the Pleistocene [10]. Molecular evidence [10,11,12] and morphometric analyses [13] suggest that *C. helleri caliginis* is not divergent enough to be considered a species, as was suggested by Grismer [14].

Snake venoms are a complex mixture of proteins and non-protein components that cause physiopathology such as edema, hemorrhage, necrosis, and diverse alterations to the skin, including blistering and dermonecrosis [15,16,17]. The venom of snakes in the Viperidae family is composed of several protein families, including snake venom metalloproteases (SVMPs), phospholipase A_2_ (PLA_2_), myotoxins (Myo), snake venom serin-proteases (SVSPs), L-amino acid oxidases (LAAOs), cysteine-rich secretory proteins (CRiSPs), C-type lectins (CTLs), disintegrins (DISs) and natriuretic peptides (NPs) [18].

Viper venoms can be characterized as type I, which have high SVMP activity and lower toxicity, and type II, that have lower SVMP activity and higher toxicity [19]. Recent studies have shown that several *Crotalus* species have β neurotoxins in their venom [20,21,22,23,24,25,26,27]. These neurotoxins directly interfere with neurotransmitter release, causing muscle paralysis [28]. The symptoms caused by proteolytic venoms are mostly local, but can occasionally cause systemic effects; commonly, the first symptoms are pain, inflammation, bleeding, and other hemostatic disorders such as platelet alterations, modification of coagulation time, and fibrinogen consumption [29], generally caused by SVMP and SVSP. Hemorrhagic effects are also common with *Crotalus* species venom.

The geographic variation of snake venom is well known in several rattlesnake species [20,30,31] as well as ontogenetic changes [30,32,33,34,35], yet the evolutionary cause of these patterns remains unclear. One of the most supported hypotheses to explain venom variation is the coevolution of venom and prey [31,36,37]. The complexity of the venom is specifically affected by phylogenetically diverse diets [38].

In Mexico, a yearly average of 3,893 snake envenomations were recorded between 2003 and 2019, resulting in 34 deaths per year. Unfortunately, no record is kept of the species causing the bite. Most bites are caused by *Bothrops asper*, *C. atrox*, *C. simus*, *C. culminatus*, and *C. mictlantecuhtli*. Most of the Mexican species have not been studied, so generating basic information on the composition of their venom will help to understand and predict the clinical syndrome. On the other hand, the evaluation of antivenom neutralization needs to be documented even when a species causes few bites [39]. While no clinical cases have been reported for *Crotalus helleri caliginis* [33], it is important to know the composition of its venom and understand the changes in the venom in a species that has been isolated for years. There is a permanent Mexican Navy presence, the island is remote, and the snake densities are high, therefore it is important to document if the Mexican antivenoms are effective in neutralizing the venom in case of an ophidian accident.

Studies on Mexican island rattlesnake venom are scarce. Glenn and Straight [40] compared lethality and enzyme activity of seven species of rattlesnakes from the Baja California Peninsula, reporting an LD_50_ of 2.6 µg/g, no β neurotoxins, no proteolytical activity, and 138 U/mg of esterase activity for *C. helleri caliginis*; Arnaud-Franco et al. [41] compared biological and biochemical activity for rattlesnakes in southern Baja California, describing an LD_50_ of 2.98, 1.56, 0.35, and 9.21 µg/g for *C. catalinensis, C. enyo enyo, C. mitchellii mitchellii*, and *C. ruber lucasensis,* respectively. On the other hand, Mackessy [42] compared the venoms of the Viridis group (in which *C. helleri caliginis* is included) using enzyme assays and electrophoretic and zymogram profiles. Furthermore, he calculated the LD_50_ and performed a mass spectrometry analysis, finding at least seven myotoxin peptides in *C. helleri caliginis* venom and reported an LD_50_ of 2.3 µg/g. However, in those experiments, only two samples of *C. helleri caliginis* venom were available, limiting the conclusions that could be made for this island subspecies, since a larger sample is required to be representative of the population.

The goals of the present work were to further investigate the biochemical and biological activities of the venom from the rattlesnake *C. helleri caliginis* and to evaluate the possible ontogenetic variation within them. In addition, the most abundant protein families of the venom were investigated, as well as the neutralization of lethal activity by two Mexican antivenoms and one experimental antivenom. As the composition of the diet of *C. helleri caliginis* on the island differs from the diet of *C. helleri helleri* on the mainland, our hypothesis was that their venom would show some differences among the two subspecies [42]. Previous studies showed that *C. helleri helleri* and other closely related species of rattlesnakes show ontogenetic changes in the venom [33,43], therefore, we also expected to see ontogenetic changes in the venom of *C. helleri caliginis.* This knowledge will be important to understand the evolutionary changes in this insular species in a geographically isolated region and their possible clinical implications.

## 2. Results

### 2.1. Crotalus helleri caliginis Samples

Venom samples of *C. helleri caliginis* were obtained from intensive fieldwork (five years of sampling 2015–2019) in South Coronado Island. A total of 210 snakes were found: 97 were male, 90 female, and 23 newborns of unknown sex. The ranges of the total body length (TBL) and weight were 212 to 825 mm and 15 to 280 g, respectively. A total of 81 individual venom samples were obtained, eight from neonates, 20 from juveniles (11 males and nine females), and 53 adults (30 males and 23 females).

### 2.2. One- and Two-Dimensional SDS-PAGE

**Figure 2:**
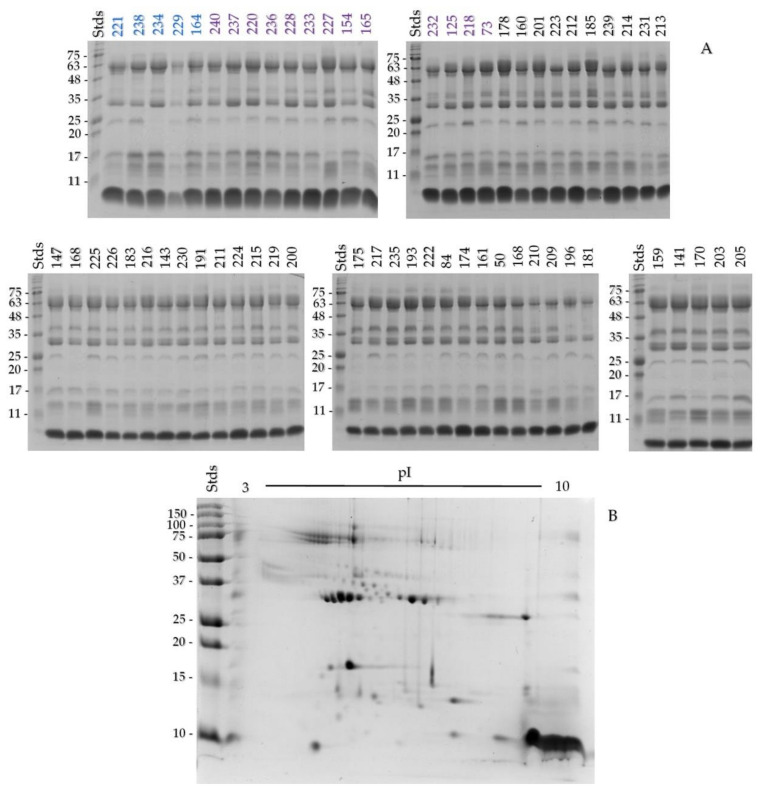
One-dimensional 15% SDS-PAGE of the neonate (blue numbers at the top), juvenile (purple), and adult (black) *Crotalus helleri caliginis* venoms under reducing conditions (**A**); 20 µg of venom were loaded per lane. Two-dimensional 15% SDS-PAGE of pooled venom under reducing conditions (**B**); a total of 150 µg was loaded.

Sixty-one venom samples of *C. helleri caliginis* were separated by SDS-PAGE (Figure 2A). Most of the samples had similar characteristics, however, specimens 168, 175, 222, 234, and 239 showed a decrease in or absence of the 25 kDa band. *C. helleri caliginis* venom was compared with *C. helleri helleri* venom (positive for crotamine and crotoxin, previously characterized in the laboratory), with other taxonomically close species, other island rattlesnakes, and crotamine isolated from *C. molossus*. The electrophoretic profile in reducing conditions was similar between *C. helleri helleri* and *C. h. caliginis*, but, in non-reducing conditions, the venoms had different profiles (Figure S1). When comparing Figure 2A and Figure S1, we can see that the newborn, juvenile, and adult venom of *C. helleri caliginis* was similar to the venom of *C. helleri helleri*.

Only a few differences in the electrophoretic profiles were observed. In order to include all possible differences, we decided to perform a pooled analysis. A pool of 55 juvenile and adults venom samples was used for the rest of the experiments. When the snake venom pool was analyzed with two-dimensional SDS-PAGE, over 70 spots were identified (Figure 2B), most of them with pI from 4–7 and MW from 10–75 kDa. There was an abundance of spots with pI above 8–10 and MW under 10 kDa, identified as crotamine. Adult and newborn venom samples were analyzed separately, and there were no differences between their profiles (Figure S2).

### 2.3. Reverse Phase HPLC

**Figure 3:**
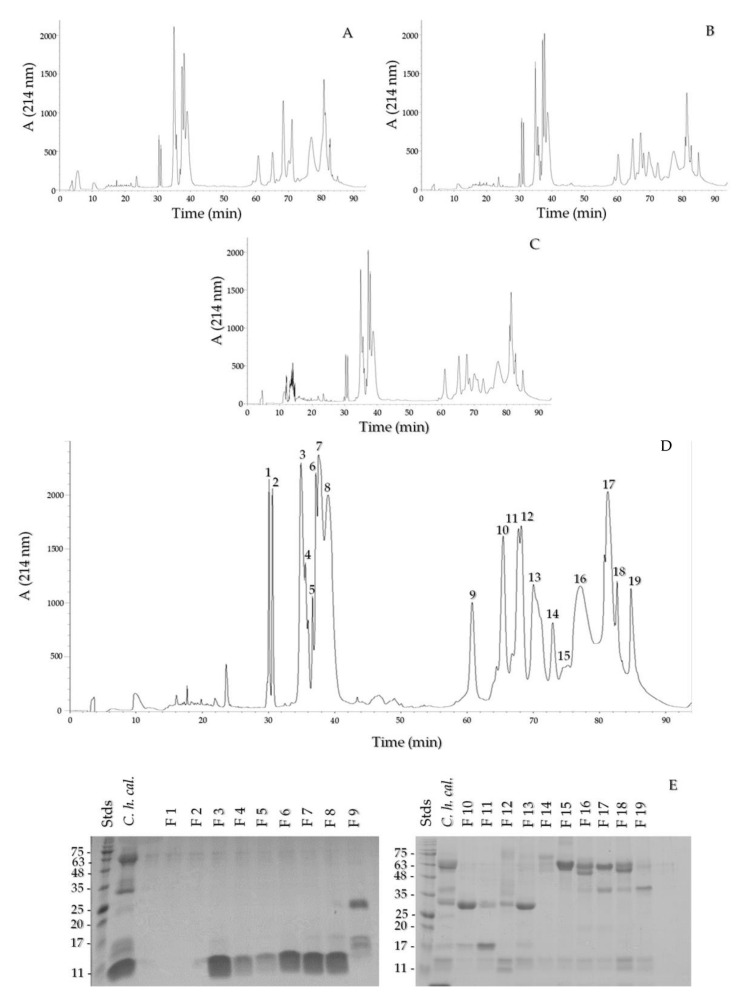
RP-HPLC profile of *C. helleri caliginis* venom on a C_18_ column. Neonate venom (**A**), juvenile (**B**), adult (**C**), and pooled venom (**D**) profiles. Pooled venom fractions on 15% SDS-PAGE under reducing conditions (**E**). Milli-absorbance units at 214 nm, A (214 nm). A total of 2 µg was loaded from each fraction.

The chromatographic profiles of a neonate venom, a juvenile venom, an adult venom, and the venom pool were analyzed (Figure 3A–D). The profiles had the same fractions in similar proportions. The fractions that eluted between 34 and 40 min were the most abundant. The pool fractions were analyzed with SDS-PAGE, which showed that the fractions from F4 to F8 presented a low MW band under 11 kDa, while fractions F9 to F13 presented mainly medium MW bands (20–35 kDa), and the last fractions presented bands higher than 40 kDa (Figure 3E). The N-terminus of F7 was obtained, which eluted in minute 38, with the sequence YKRCHKKGGHCFPKTVICLPPSSDFGKMDCR, matching 100% with a crotamine from *C. helleri helleri* (AEU60012.1) from a venom gland, Protein Database, NCBI, and 100% with peptide C of *C. helleri helleri* venom reported by Maeda and Tamiya [44]. The masses of the 3–8 peaks are 4995.11 (and 4883.58), 5208, 5095.79 (and 5208), 5167.69, 5094.1 (and 4983.22), and 4981.6 Da, respectively (Figure S3).

### 2.4. SVMP, SVSP, and Crotamine Detection by Western Blot

**Figure 4:**
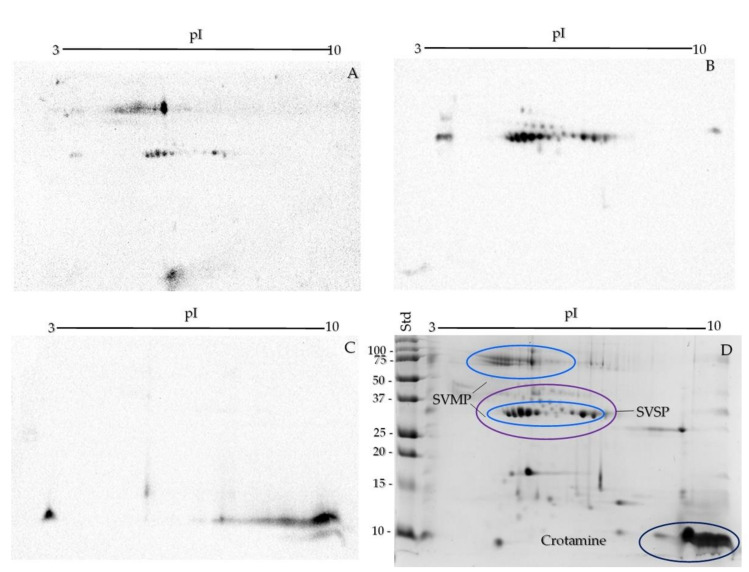
Western blot of the crude venom of *C. helleri caliginis* on two-dimensional SDS-PAGE using antibodies against the three main venom protein components: SVMP (**A**), SVSP (**B**), and crotamine (**C**). The SDS-PAGE (**D**) was 15% polyacrylamide and in the 3–10 isoelectric point (pI) range, 50 µg of venom were loaded for the Western blot and 150 µg for the two-dimensional SDS-PAGE.

Western blot was used for the identification of the protein families, with anti-SVMP, SVSP [30,45], and crotamine antibodies [46] (Figure 4A–C). SVMPs were observed with pI between 4 and 7 and MW around 75 and 30 kDa (Figure 4A). Most of the spots around 25–50 kDa and a pI range between 4 and 7 belong to the SVSP family, though some of the spots showed the same pI and MW as the SVMP family (Figure 4B). To obtain a better resolution in the area where SVMP and SVSP families appear, a two-dimensional 12% SDS-PAGE with a pI range from 4 to 7 was run (Figure S4). Crotamine was represented by a large patch around 10 kDa and in the 8–10 pI range (Figure 4C). Additionally, a protein with an acidic pI was recognized with this serum, probably a PLA_2_ that was cross-recognized, as previously documented [46].

### 2.5. Size-Exclusion Chromatography and Crotamine Purification

**Figure 5:**
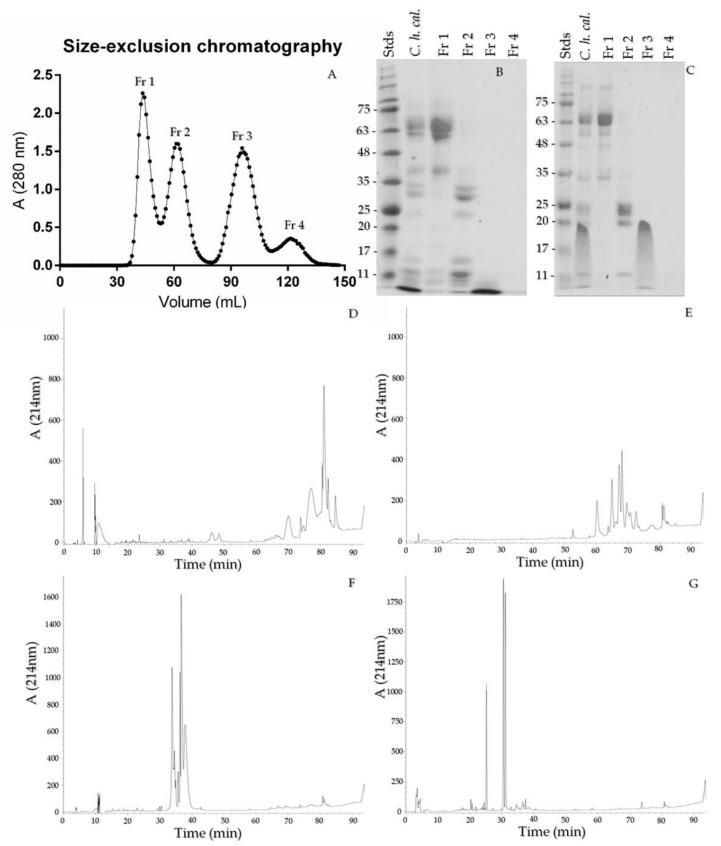
Size-exclusion chromatography profile of the crude venom of *C. helleri caliginis* (**A**), 15% SDS-PAGE under reducing (**B**) and non-reducing conditions (**C**). RP-HPLC profile of *C. helleri caliginis* SEC fractions 1 to 4 (**D**–**G**, respectively). A total of 20 µg of *C. helleri caliginis* venom and 5 µg of every fraction was loaded. A total of 50 mg of *C. helleri caliginis* venom was loaded in the Sephadex columns.

Four fractions were obtained by size-exclusion chromatography; every fraction was run on 15% SDS-PAGE under reducing and non-reducing conditions and analyzed with RP-HPLC (Figure 5A–C). Fraction 1 (F1) presented high MW bands and a ~35 kDa band that eluted in minutes 70 to 94; F2 contained 20–30 kDa proteins with retention times between 60 and 75 min; F3 consisted only of crotamine with MW under 11 kDa; and in fraction F4, no protein bands were clearly observed, this is due to the fact that this fraction probably elutes non-protein components and small peptides (Figure 5G) that are not observed under the conditions of our gel (Figure 5 D–G).

### 2.6. Mouse Phrenic Nerve–Hemidiaphragm Preparation

**Figure 6:**
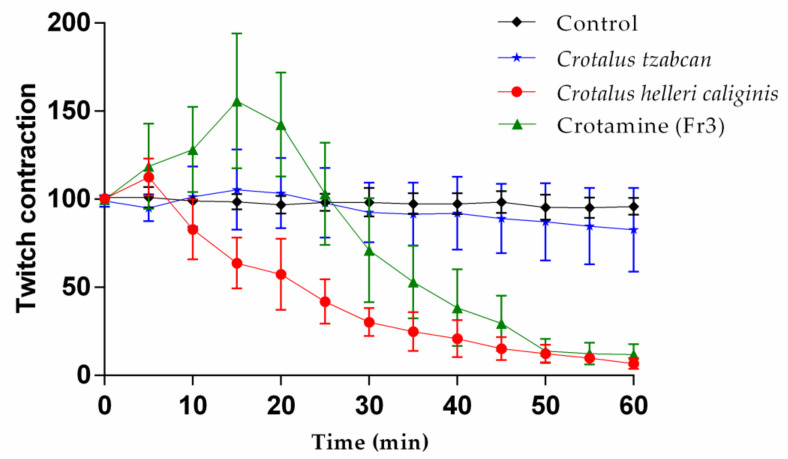
Neuromuscular effect of *C. helleri caliginis* crude venom and crotamine (Fr3) on indirectly evoked muscle twitches. The final crude venom concentration was 5 μg/mL and 2 μg/mL for crotamine. *Crotalus tzabcan* venom without crotamine was used as a positive control and Tyrode solution as a negative control. Points represent the mean ± SD of three replicates. The data were normalized using 100% the average force (g) of directly evoked twitches 5 min before venom or toxin addition.

*Crotalus helleri caliginis* total venom causes inhibition of indirectly evoked muscle contractions, with a T_90_ of 45 min, as shown in Figure 6; we also observed an increase in the twitch force in the first 5 min after venom addition. With the purified crotamine (Fr3), the increase lasted 15 min and the T_90_ was 55 min. In the interval between stimuli (every five seconds), the muscle showed fasciculations in their first 20 min with crotamine (data not shown). The increase in the twitch tension with the crude venom was 112.5% at the highest point (minute 5) and 155.8% at minute 15 with the purified crotamine (Fr3). Directly evoked muscle twitches were also completely inhibited by the end of the experiment. *Crotalus tzabcan* venom without crotamine (unpublished data) was used as a positive control and Tyrode solution as a negative control.

### 2.7. Biological, Biochemical, and Lethal Activity of C. helleri caliginis Venom

The biological and biochemical activities of *C. helleri caliginis* compared to *C. helleri helleri* and two Mexican island rattlesnakes (*C. catalinensis* and *C. pyrrhus muertensis*) are summarized in Table 1. The crude venom of *C. helleri caliginis* showed a similar lethal dose in rats, mice, and chickens, with 0.54 (0.5–0.6), 0.58 (0.5–0.65), and 0.62 (0.55–0.7) µg/g, respectively. The most lethal fraction of *C. helleri caliginis* venom in mice was Fr1, with an LD_50_ of 0.64 (0.45–0.72) µg/g compared to 2.22 (2–2.45) and 2.93 (2.7–3.1) µg/g of Fr2 and Fr3, respectively; Fr4 showed no lethal activity with 5 µg/g. The *C. catalinensis* venom showed an LD_50_ of 1.47 (1.4–1.5) µg/g and it was 1.16 (1.0–1.3) µg/g for *C. pyrrhus muertensis* venom.

The phospholipase and proteolytic activity tests showed lower activity in the *C. helleri caliginis* and *C. helleri helleri* venoms (28 ± 3, 0.53 ± 0.06 and 32 ± 2.5, 0.94 ± 0.05 U/mg, respectively) compared to the island rattlesnake venoms of *C. catalinensis* and *C. pyrrhus muertensis* (145 ± 14, 1.4 ± 0.07 and 165 ± 12.5, 1.98 ± 0.16 U/mg, respectively). The four venoms showed fibrinogenolytic activity; *C. helleri caliginis* venom acts on the α chain and the other three venoms degrade α and β chains. Only *C. helleri helleri* tested positive for crotoxin, and the three island rattlesnakes were negative by ELISA.

### 2.8. Zymogram on SDS-PAGE Copolymerized with Gelatin

**Figure 7:**
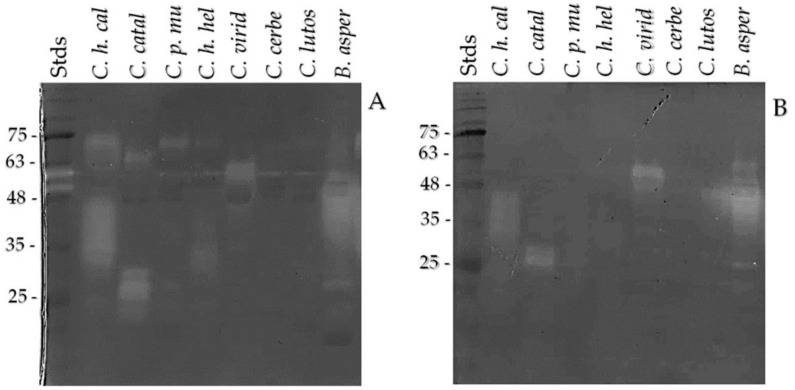
Zymogram of *Crotalus helleri caliginis venom* compared to that of *C. catalinensis*, *C. pyrrhus muertensis*, *C. helleri helleri*, *C. viridis*, *C. cerberus*, and *C. lutosus*. *Bothrops asper* venom was used as a control of proteolytic activity. Crude venoms (**A**) and venoms treated with EDTA 5 mM (**B**). A 12% native PAGE copolymerized with gelatin; 5 µg of venoms were loaded per lane.

The zymogram showed the presence of proteins with proteolytic activity (Figure 7A). We observed the proteolytic activity in *C. helleri caliginis* venom (Figure 7, lane 2), showing two zones of activity, the first one below 75 kDa and the second between 30 and 48 kDa. *C. catalinensis* venom degraded the gelatin at 65 and 25–30 kDa, while *C. pyrrhus muertensis* venom only showed degradation around 70 kDa. *C. helleri caliginis* venom was similar to *C. helleri helleri* in the electrophoretic profile, but the proteolytic activity in gelatin was different, showing activity at 35 and 70 kDa, whereas *C. viridis* venom had activity between 48 and 63 kDa. *Crotalus cerberus* and *Crotalus lutosus* venom showed no activity. *Bothrops asper* venom was used as control for the high proteolitic activity on the venom.

When the venoms were treated with EDTA, an inhibitor of SVMP (Figure 7B), the *C. helleri caliginis* venom only showed activity in the 30–48 kDa zone. The same activity zone for *C. viridis* venom (48–63 kDa) appeared with EDTA treatment and without. The control venom used (*B. asper*) displayed the same activity zone after the EDTA treatment.

### 2.9. Neutralization Capacity of Mexican Commercial Antivenoms

The results of the neutralization of lethality with two commercial antivenoms and an experimental antivenom are summarized in Table 2. Antivipmyn^®^ showed the best neutralization potency (6.4 mg·AV/mg·V) against *C. helleri caliginis* venom, neutralizing 789 lethal doses per vial. A greater concentration of Faboterapico polivalente antiviperino^®^ was needed (29 mg·AV/mg·V) to neutralize the same amount of venom than Antivipmyn^®^. However, since the vial contains more protein, it can neutralize 875 LD_50_/vial. Inoserp^®^ antivenom only neutralizes 324 LD_50_/vial. None of the antivenoms tested could neutralize the spastic paralysis symptom in mice caused by crotamine.

## 3. Discussion

*Crotalus helleri caliginis* was possibly isolated from the continent during the Pleistocene [10], however, it is scarcely divergent at the molecular level [13] and the venom components remain similar to *C. helleri helleri*. *C. helleri caliginis,* possibly recently, changed its natural diet from mainly mammals (*Peromyscus maniculatus assimilis*) to mostly lizards (*Elgaria nana* and *Plestiodon skiltonianus*) and birds (*Larus occidentalis* and *Ptychoramphus aleuticus*), probably caused by human incursion on the island, which caused a population decline of *Peromyscus maniculatus assimilis*. Perhaps the divergence time has not been enough to establish big differences in the proteomic venom profile between these subspecies.

An ontogenetic venom change has been documented in *Crotalus helleri helleri* [33], consisting of venoms with low proteolytic activity and high PLA_2_ activity in neonates and juveniles, that then decrease their PLA_2_ activity and increase their proteolytic activity as adults. Other members of the Viridis group also show ontogenetic changes in the LD_50_ [43], with juveniles being more lethal than adults [32], and shifting from SVMP-rich venom to a myotoxic venom [34]. There is no ontogenic change in *C. helleri caliginis* venom, given that the five neonate, 13 juvenile, and 43 adult venoms did not show any variation in the electrophoretic profile (Figure 2A). The chromatograms of the neonate, juvenile, and adult venoms displayed the same peaks (Figure 4A–D). This lack of ontogenetic change could be a consequence of the absence of a diet shift during *C. h. caliginis* development [9], while *C. helleri helleri* changed its diet from lizards as neonates and juveniles to mammals and birds as adults [33].

Variation in venom composition is affected by diet, particularly in dietary specialists [31,36,37,47]. However, it has been suggested that [47] gene flow may be more likely to drive changes in venom composition in dietary generalists. Although we found the venom of the subspecies to be similar to its mainland counterpart, it is possible that the recent divergence of these lineages is insufficient for subsequent venom divergence. In our analysis, we found that the venom of newborns, juveniles, and adults of *C. helleri caliginis* remains unchanged; moreover, we also found that the venom of the three stages of *C. helleri caliginis* is more similar to *C. helleri helleri* adult venom than to the venom of juveniles or newborns. These two observations are partially explainable with results from the literature: first, the lack of variation in venom throughout the lifetime of *C. helleri caliginis* might be simply due to the lack of changes in its diet, something that we also documented with our unpublished observations in the field. Second, we expected that the venom from *C. helleri caliginis* would be more similar to the venom of newborn and juvenile specimens of *C. helleri helleri*, which show a diet more similar to the island rattlesnakes. However, this was not the case, which may be due to the fact that the island subspecies and mainland adults both have more generalist diets consisting of a variety of prey types.

The geographic venom variation in *C. helleri* is well known [20,32,34,48]. *C. helleri caliginis* venom is similar to *C. helleri helleri,* as stated by Mackessy [42], but it is different from those venoms containing crotoxin, as Jurado et al. [48] showed. *C. helleri caliginis* and *C. helleri helleri* venoms are similar not only in the one-dimensional SDS-PAGE profile (Figure S2), but the two-dimensional SDS-PAGE of *C. helleri caliginis* venoms shares some proteins with *C. helleri helleri* venom (San Jacinto Mountain Range distribution), with Jurado et al. [48]**,** comparing results of the Ch15 two-dimensional SDS-PAGE venom, finding that the spots from 25–50 kDa and 4–7 pI are also similar in the two subspecies.

The most abundant protein families in *Crotalus* venoms are PLA_2_, SVMP, and SVSP, at 60–80% of the total venom [18], while the rest of the components are usually less abundant. *C. helleri caliginis* venom exhibits a high percentage of SVMP, SVSP, and crotamine: the Western blot of the two-dimensional SDS-PAGE recognized 27 spots corresponding to SVMP, 26 spots of SVSP, and two big spots of crotamine (Figure 3C–D). Eighteen of the 73 spots were not assigned to a snake venom family. Using the PDQuest software (Biorad), SVMP, SVSP, and crotamine were found to comprise ~65–75% of the proteins separated by two-dimensional SDS-PAGE of *C. helleri caliginis* venom, with crotamine being the most abundant component (30–35%), similar to what is reported by Maeda et al. [44] for *C. helleri helleri*.

Crotamine causes muscle contracture [49,50] and hind limb paralysis in mice and rats. Rigid paralysis was observed when performing lethality tests on mice, rats, and chickens. This toxin likely helps to momentarily paralyze the prey, gaining a few seconds while the rest of the toxins take effect and kill the prey. We obtained 19 proteins with RP-HPLC, where the first peaks that elute from 33–40 min were different crotamine isoforms. Crotamine has been reported for *C. helleri caliginis* and *C. helleri helleri* venoms [19,51], and we obtained at least six peaks (possible isoforms); each peak was injected into mice, and all produced rigid paralysis in the hind legs of mice. These fractions represent approximately 30–35% of the total venom (Figure 3E–F). The peak 7 sequence (YKRCHKKGGHCFPKTVICLPPSSDFGKMDCR) is 100% identical to the peptide C sequence reported by Maeda et al. [44] in the *C. helleri helleri* venom. The peak 8 sequence is similar to peak 7, with only changes in the amino acid residues 15, 16, and 19 (YKRCHKKGGHCFPKEKICIPPSSDFG).

Size-exclusion chromatography was used with Sephadex G75 to isolate crotamine (Figure 5) from other components, as Toyama [52] and Chang and Tseng [53] did with *Crotalus durissus terrificus* venom. In mouse phrenic nerve neuromuscular preparations, crotamine induced an 118% increase in the contraction force of indirectly evoked twitches in the first 5 min and a maximum of 156% at 15 min (Figure 6), the same as Lima et al. [54] and Chang and Tseng [53] who reported the increase in twitch contraction after crotamine addition. The full venom of *C. helleri caliginis* and the isolated crotamine induce a complete inhibition of muscle twitches with T_90_ of 45 and 55 min with 5 and 2 μg/mL, respectively. These venoms show a significant potential to induce rigid paralysis. Indeed, the *C. durissus terrificus* crotamine has an LD_50_ of 1.5–3.0 μg/g [55,56], and it is 1.96 μg/g for the *C. helleri helleri* [44] crotamine, more toxic than *C. helleri caliginis* crotamine at 2.93 (2.7–3.1) µg/g. Further studies are needed to determine the exact mechanism of inhibition of muscle twitches and whether it is caused by crotamine alone or by a combination of this toxin with other venom components.

In 1985, Glenn and Straight [57] were the first to report the LD_50_ of *C. helleri caliginis* as 2.6 µg/g. Twenty-five years later, Mackessy [42] reported an LD_50_ of 2.3 µg/g. We show an LD_50_ in rats, mice, and chicken under 1 µg/g (0.54, 0.58, and 0.62 µg/g, respectively), and the difference in our study compared to the previous ones is the number of samples we had available. We used a venom pool from 55 individuals, while the previously mentioned authors only used two venom samples. A loss of lethality was observed when the venom was resuspended in PBS and stored at 4 °C for a prolonged time (1 week), but this loss of activity did not affect the electrophoretic profile.

A recent study by Dowell et al. [58] demonstrated that the neurotoxic PLA_2_ (crotoxin) appeared in *C. helleri helleri* venom through hybridization with *C. scutulatus* near the San Jacinto mountains. There is no report of crotoxin in other *C. helleri helleri* populations in California [20,59], showing that the common ancestor of *C. helleri caliginis* and *C. helleri helleri* might have been crotoxin negative. No crotoxin isoforms were found in *C. helleri caliginis* venom (Table 1). Additionally, *C. helleri helleri* and *C. helleri caliginis* venoms showed a low proteolytic and PLA_2_ activity (0.94 ± 0.05 and 32 ± 2.5; 0.53 ± 0.06 and 28 ± 3 U/mg, respectively) as opposed to the other two island rattlesnakes (*C. catalinensis*, 1.4 ± 0.07 and 145 ± 14 U/mg; *C. pyrrhus muertensis*, 1.98 ± 0.16 and 165 ± 12.5 U/mg). Glenn and Straight [57] reported no proteolytic activity of *C. helleri caliginis* venom on Remazol brilliant blue-hide powder azure, while we report a low proteolytic activity on azocasein.

The results on zymograms showed a difference in the proteolytic activity on azocasein, where the *C. helleri caliginis* venom has a higher activity than *C. catalinensis* and *C. pyrrhus muertensis*. We identified two activity zones of *C. helleri caliginis*: between 60 and 75 kDa and between 30 and 50 kDa, the same as Mackessy [42], but we proved that the activity is not only caused by SVMP, but also by SVSP on gelatin using EDTA to inhibit SVMP [60].

The three Mexican antivenoms (Antivipmyn^®^, Faboterapico polivalente antiviperino^®^, and Inoserp^®^) neutralized the lethal activity of *C. helleri caliginis* venom, with Antivipmyn^®^ showing the best neuralization (6.4 mg AV/mg V), followed by Inoserp^®^ and Faboterapico polivalente antiviperino^®^ (21.7 and 29 mg AV/mg V, respectively). However, neither of the three antivenoms could neutralize the crotamine effect, similar to what was reported by Borja et al. [30] for *Crotalus molossus* venom, where the antivenoms did not recognize the myotoxins in the Western blot and in vivo testing. These results suggest that Mexican pharmaceuticals need to improve antivenoms using crotamine-positive venoms to neutralize the crotamine effects, the main component of the *C. helleri caliginis* venom.

Crotamine is an important component of Mexican rattlesnake venom [30,61]. It is found in significant proportions in juvenile specimens of *C. molossus nigrescens* [30], *C. tzabcan*, and *C. culminatus* [61], decreasing in proportion ontogenetically. It has also been documented in adult specimens of *C. scutulatus* and *C. scutulatus salvini* [62,63], and it is very likely present in other species or populations of Mexican rattlesnakes not studied to date. As this toxin can cause significant damage to the muscle [54], its neutralization by antivenoms is necessary. Furthermore, it has been shown that antivenoms are unable to neutralize the crotamine effect in mouse models. This is caused by its low immunogenicity and probably by its low proportion or absence in the venoms that are used as immunogens for the production of antivenoms. Therefore, it is necessary to include crotamine-rich venoms as immunogens for the production of antivenoms.

## 4. Conclusions

*C. helleri caliginis* venom has no ontogenetic changes and is very similar to the crotoxin-negative *C. helleri helleri* adult venoms. Over 70 proteins were separated, approximately 50 were identified as SVMP, SVSP, and crotamine, with crotamine being the main component (35%) of *C. helleri caliginis* venom. Additionally, the venom causes a fibrinolytic effect on the α chain, but the main effect was the muscle paralysis caused by crotamine. The venom has more than six isoforms of crotamine and more studies are needed to identify and characterize them in detail.

The Mexican antivenoms neutralize the lethality of the *Crotalus helleri caliginis* venom, but they cannot neutralize the effect of crotamine, the main component of the *C. helleri caliginis* venom and abundant in several Mexican rattlesnakes. Therefore, we recommend that antivenom manufacturers make improvements to the product by including venom from crotamine-positive specimens.

## 5. Materials and Methods

### 5.1. Ethics Statement

We followed the guidelines by the Ethics Committee of the Autonomous University of Aguascalientes which is compatible with NOM-062-ZOO-1999 for the use of animals (rats, mice, chickens). We obtained collection permits from the Dirección General de Vida Silvestre de la Secretaría del Medio Ambiente y Recursos Naturales (DGVS-SEMARNAT) (SGPA/DGVS/00288/19, UG/211/00145/2019), scientific activities permission from the Comisión Nacional de Áreas Naturales Protegidas (CONANP), and disembarking permits from the Secretaría de Gobernación (SEGOB) and Secretaría de Marina (SEMAR), all issued to Jesús Sigala-Rodríguez.

### 5.2. Venoms

Snakes were captured on South Coronado Island, measurements were taken (age, sex, rattle segments, snout–vent length, tail length, and weight), and venom was extracted prior to release in the same place. The venom extraction was carried out on the island, and the venoms were stored in a liquid nitrogen tank, then thawed, centrifuged, and lyophilized in the laboratory, following the same process we have used in the past [64]. Lyophilizing the venom would prevent further degradation during the five-year period of the study. Each snake was marked with a pit-tag to recognize it in case of subsequent recaptures. The venom was centrifuged to eliminate cellular debris and non-soluble proteins, lyophilized, and stored at −70 °C until use. The neonate samples were obtained from snakes that only had a rattle button and no additional rattle segments, juveniles were those below 450 mm of total length, with a triangular rattle, and specimens were considered adults when larger than 450 mm with a non-tapered rattle. Only 61 venoms of 81 specimens were analyzed, 20 venom samples were not analyzed individually because we mixed them before the experiments to use as venom pools.

### 5.3. Protein Quantification

We weighed between 5 and 10 mg of lyophilized venom, resuspended it in PBS, and then quantified it by a Bradford protein assay (Bio-Rad), following the instructions from the manufacturer with bovine serum albumin (BSA) as a standard. This method measures the presence of basic amino acid residues, arginine, lysine, and histidine. All the samples, including the standard, were measured in triplicate. *Crotalus helleri helleri* venom was bought from the National Natural Toxins Research Center (NNTRC). The venoms of *Bothrops asper, C. catalinensis, C. cerberus, C. lutosus, C. pyrrhus muertensis, C. tzabcan*, and *C. viridis,* came from the venom bank of the Herpetario Cantil, Instituto de Biotecnología, UNAM.

### 5.4. One-Dimensional SDS-PAGE

One-dimensional SDS-PAGE was performed using a 15% polyacrylamide gel on a Mini-Protean Tetra cell (Bio-rad, Hercules, CA, USA). Twenty micrograms of each venom were dissolved in sample buffer (65.8 mM Tris-HCL, pH 6.8, 26.3% glycerol, 2.1% SDS, 0.01% bromo-phenol blue) with 5% of β-mercaptoethanol for reducing conditions. Each sample was boiled for 5 min and run at 100 V. The gels ran under non-reducing conditions, had no β-mercaptoethanol, and were not boiled. Gels were stained with Coomassie Brilliant Blue R-250 (Bio-Rad, Hercules, CA, USA) for 2 h and rinsed with 40% methanol and 10% acetic acid. Dual Color Standards (Bio-Rad, Hercules, CA, USA) were used as standard molecular mass markers.

### 5.5. Two-Dimensional SDS-PAGE

The ReadyPrep^TM^ 2-D Starter Kit of BioRad was used following the instructions from the manufacturer. We mixed 150 µg of *C. helleri caliginis* venom with the ReadyPrep Rehydration/Sample Buffer in a final volume of 125 µL, then loaded the sample on the tray channel, immediately put the 7 cm ReadyStrip^TM^ IPG strip pH 3–10 or 4–7 side down onto the protein sample and covered it with 2 mL of mineral oil to avoid evaporation. The strips were rehydrated for 12 h on the PROTEAN i12^TM^ IEF system and were run for 15 min at 250 V, 60 min at 4000 V, and 3–4 h at 15,000 V/h at 4000 V. Finally, the strips were held at 500 V.

Afterwards, the oil from the strip was drained and it was cleaned with TGS running buffer, then placed in an incubation shaker with Equilibration Buffer I for 10 min (375 mM Tris-HCl, pH 8.8, 6 M urea, 2% SDS, 2% DTT) and a further 10 min with Equilibration Buffer II (375 mM Tris-HCl, pH 8.8, 6 M urea, 2% SDS). Finally, the strip was placed with the gel side up on the backplate of the 15% SDS-PAGE gel and covered with agarose to maintain contact between the stripping gel and the SDS-PAGE gel. The gel was run for 2 h at 100 V and stained with Coomassie Brillant Blue R-250 for 30 min and rinsed with 40% methanol and 10% acetic acid. The imaging system used was a ChemiDoc XRS+ System.

### 5.6. Western Blot

A total of 50 µg of *C. helleri caliginis* venom was run on a two-dimensional SDS-PAGE as previously described, using a 0.45 µm nitrocellulose membrane and a Trans-Blot^®^ SD Semi-Dry Transfer Cell (BioRad) to transfer the proteins using TGS with methanol 20% as a transfer buffer. After transference at 20 V for 25 min, the membrane was blocked with 5% non-fat dry milk (blotting-grade blocker, BioRad, Hercules, CA, USA) diluted in TBST (0.05 M Tris-HCl, 0.15 M NaCl + 0.05% of Tween-20, pH 7.4) for 3 h. The membrane was rinsed with 20 mL of TBST 4x for 15 min and incubated by gentle shaking with the primary antibody (anti-SVMP, anti-SVSP, or anti-crotamine) raised in rabbits; 5 µg/mL of primary antibody in a final volume of 10 mL with TBST for 2 h at room temperature (~25 °C). The membrane was rinsed again four times with 20 mL of TBST for 15 min, and then incubated with the second antibody, goat anti-rabbit conjugated with alkaline phosphatase (1:5000), for 2 h. The membrane was rinsed four additional times, and then we used the Clarity Western ECL (BioRad, Hercules, CA, USA) for 5 min. Finally, the ChemiDoc XRS+ System was used to develop the membrane with the Chemi UV Transilluminator protocol with an exposure of 1–10 s.

### 5.7. Reverse Phase HPLC

For the RP-HPLC, the Agilent 1100 chromatography system with a C18 reverse phase analytical column (Vydac ^®^, Deerfield, IL, USA, 218 TP 4.6 mm × 250 mm) was used for the separation of venom proteins. A total of 1 mg of venom was dissolved in 1 mL of ultrapure water with 0.1% trifluoroacetic acid (TFA). The flow rate was 1 mL/min, and the elution profile was obtained by the percentage of solution B (acetonitrile with 0.1% TFA), as follows: 0% B for 5 min, 0–15% B for 10 min, 15 to 45% for 60 min, 45–70% of B for 10 min, and finally 70% for 4 min. Twelve different venoms of *C. helleri caliginis* (one newborn, three juveniles, five adults, and three captive adults) were run and compared for any differences. After no differences were seen in the profile, a pool of 55 venom samples was used and run with RP-HPLC. All peaks were collected manually and dried by vacuum centrifugation (SpeedVac, Thermo, Asheville, NC, USA). The peaks were run on a 15% SDS-PAGE gel under reducing conditions.

### 5.8. Molecular Mass and N-Terminal Sequence Determination of Crotamine-Like Myotoxins

The masses of the crotamine isoforms (fractions 3–8, by RP-HPLC) of *C. helleri caliginis* venom were determined using mass spectrometry with electrospray ionization (ESI-MS) on an LCQ Fleet Ion Trap Mass Spectrometer. Amino-terminal sequencing of two crotamine isoforms was determined by automated Edman degradation on a PPSQ-31A Protein Sequencer (Shimadzu, Tokyo, Japan) as previously described by Borja et al. [30].

### 5.9. Size-Exclusion Chromatography and Crotamine Purification

A 196 cm long and 0.9 cm diameter column glass was used, packed with Sephadex G-75 (Sigma) and PBS as a buffer with a flow of 15 mL/h. One milliliter (50 mg/mL) of the sample was added to the column. Samples of 1 mL per tube were collected, the fractions were immediately placed on ice, and the LD_50_ measures were immediately performed; afterwards, the samples were lyophilized and stored at −20 °C until we used them. We ran electrophoresis in reducing and non-reducing conditions with the fractions and loaded an RP-HPLC to identify the section of the crude venom present per sample.

### 5.10. Mouse Phrenic Nerve–Hemidiaphragm Neuromuscular Preparation

To observe the crude venom and crotoxin effects on muscle contraction, a mouse phrenic nerve–hemidiaphragm neuromuscular preparation, originally described by Bülbring [65], was used. CD-1 mice of 30–35 g were euthanized with CO_2_ inhalation, and the left phrenic nerve with its diaphragm muscle was excised. The tissue was mounted as described by Neri-Castro et al. [66] in a 20 mL organ bath with Tyrode solution (0.4 mM NaH_2_PO_4_, 2.7 mM KCl, 1.8 mM CaCl_2_, 1.0 mM MgCl_2_, 137 mM NaCl, and 11.9 mM NaHCO_3_, pH 7.2) with 11 mM glucose, at 37 °C. The bath was constantly bubbled (95% O_2_, 5% CO_2_) to oxygenate the tissue. The contractile response was recorded using an isotonic force transducer (50 g, BioPac Systems UIM100 with TSD125C signal amplifier).

For the nerve stimulus (indirect stimulation), we used two platinum electrodes in contact with the phrenic nerve. The muscle was stimulated with two platinum electrodes, one in contact with the muscle tissue and the other submerged on the Tyrode solution (direct stimulation). We used supramaximal voltage pulses with a duration of 0.1 ms and 0.2 PPS (pulses per second). Then, 10 μM δ-tubocurarine, a reversible blocker of neuromuscular transmission, was used to make sure the nerve and muscle stimulations were completely independent of each other. We washed the tissue three times to remove the blocker until the original twitch tension was recovered.

Finally, a 5 µg/mL final concentration of venom or 2 µg/mL of crotamine (fraction 3) was added to the Tyrode solution and left in contact with the tissue for 1 h. The results were exported from Biopac Student Lab 4.1.2 to GraphPad Prism 6.0 for the data analysis.

### 5.11. Median Lethal Dose (LD_50_)

Since we documented that the natural diet of *C. helleri caliginis* is based mainly on reptiles, birds, and rodents, we decided to compare the lethal dose in two of these groups. Lyophilized venom was re-suspended in PBS and injected into the caudal vein of CD1 mice (18–20 g) and Wistar rats (280–300 g), and into the jugular vein of chickens. We did not check for sex differences. We also used the fractions obtained by size-exclusion chromatography. We used six different doses on a group of three mice, and the final injected volume was 0.5 mL. The logarithm of the venom injected was plotted against the mortality percentage in the program GraphPad Prism 6.0, using non-parametric methods (Sigmoidal dose–response), as described in Borja et al. [30].

### 5.12. PLA_2_ Activity

PLA2 activity was determined through a titrimetric assay. We used a 10% egg yolk solution (0.1 M NaCl, 0.01 M CaCl_2_, 0.5% Triton-100, and 10% egg yolk) as substrate in a final volume of 500 µL of the solution adjusted to 8.05 pH with 50 mM NaOH. The solution was under constant N_2_ bubbling and stirring. We added 1 µL of the venom to a final concentration of 1 mg/mL, and when the pH was 7.99, we added ~3 µL of 50 mM NaOH to maintain the pH between 8.00 and 8.05 [67]. We repeated the procedure to complete 5 measurements. Units of enzymatic activity (U) were defined as μmoles of NaOH consumed per minute, and the results were reported in units per milligram of venom (U/mg). All the venoms were tested in triplicate, using *Micrurus* venom as a positive control.

### 5.13. Proteolytic Activity

The azocasein method to evaluate proteolytic activity was used. The azocasein was dissolved in a final concentration of 10 mg/mL in standard solution (50 mM Tris-HCl, 150 mM NaCl and 5 mM CaCl_2_). A total of 20 μg of venom were added to 100 µL of the final azocasein solution and incubated 30 min at 37 °C. After incubation, the reaction was stopped by adding 200 μL of 5% trichloroacetic acid and centrifugated at 13,500× *g* for 5 min. One hundred and fifty microliters of the supernatant were taken and added to a 96-well plate with 150 μL of 500 mM NaOH. Finally, the samples were read at 450 nm. Units of enzymatic activity (U) were defined as a change of 0.2 in the absorbance of the sample per minute [68].

### 5.14. Fibrinolytic Activity

For the fibrinolytic assay, 100 µg of lamb fibrinogen were incubated with 10 µg snake venom for 40 min at 38 °C. The samples were run on 12.5% SDS-PAGE under reducing conditions using 5 µg fibrinogen without venom as a negative control. The gel was stained and de-stained to observe the bands of the α, β, and γ chains and identify which band had been degraded by the venom.

### 5.15. Detection of Crotoxin by ELISA

An ELISA assay was run as previously described [24,66,69]. ELISA plates (96-well plates, Nunc MaxiSorp™) were sensitized with 100 μL of 1 μg/mL 4F6 monoclonal antibody for 1 h. After washing, the wells were blocked for 2 h with gelatin (200 μL of 0.5% gelatin in 50 mM Tris, 0.2% Tween 20, pH 8.0). A standard curve with crotoxin at a starting concentration of 5 μg/mL and 1:3 consecutive dilutions were generated. Venom samples of *C. helleri caliginis, C. helleri helleri, C. catalinensis,* and *C. pyrrhus muertensis* (100 µL) were loaded in each well (final concentration of 5 µg/mL) with subsequent 1:3 serial dilutions and the plate was washed three times. Then, 100 µL of a polyclonal rabbit anti-crotoxin antibody (2 µg/mL) was loaded, washed three times, and HRP-conjugated goat anti-rabbit IgG was added (diluted 1:4000) for 1 h at 37 °C. Finally, 100 µL of the revealing buffer 2,2-azino-bis (3-ethylbenzothiazoline-6-sulphonic acid/H_2_O_2_) were added to each well; after 20 min at room temperature, the absorbance was read at 405 nm on a microplate spectrophotometer (Magellan R).

### 5.16. Zymograms

For the proteolytic activity on gel, 12.5% SDS-PAGE co-polymerized with gelatin was used with a final concentration of 1.5 mg/mL. Five micrograms of venom with and without treatment of EDTA 5 mM that ran in a non-reducing condition were loaded. The gel was then incubated in 50 mL of Tris-HCL buffer (0.1 M, pH 8, 5% Triton X-100) for 1 h. Then, the gel was incubated for 1 h in 50 mL of Tris-HCL buffer (0.1 M, pH 8, 0.05% Triton X-100) and finally for an additional hour in Tris-HCL buffer (0.1 M, pH 8). All the incubations were carried out at room temperature under gentle shaking. The gel was left for 10 h in a wet chamber, stained with Coomassie Brilliant Blue R-250 (Bio-Rad, Hercules, CA USA) for 20 min, and rinsed out with 40% methanol and 10% acetic acid.

### 5.17. Antivenom Neutralization

Three commercially available Mexican antivenoms were used for this experiment: Antivipmyn^®^ manufactured by Bioclon (Lot. B-8K-31), Faboterapico polivalente antiviperino^®^ manufactured by Birmex (Lot. FV044A), and Inoserp^®^ manufactured by Inosan (Lot. 8805181002). They were tested by intravenous injection of 3 LD_50_ preincubated with different volumes of antivenom in groups of three 18–20 g mice per dose. Deaths were recorded 24 h after inoculation, and the results were analyzed as described for the determination of LD_50_ [70].

## Figures and Tables

**Table 1:** Comparative table of *C. helleri caliginis, C. helleri helleri,* and other island rattlesnake venoms on their biological, biochemical, and toxicological activities as well as identification of crotoxin homologs by ELISA.

Species	Geographic Location	LD_50_ (µg/g)			PLA_2_ (U/mg) *	Proteolysis (U/mg) *	Fibrinolytic Activity **	Crotoxin Detection
		Rat	Mouse	Chicken				
*C. helleri caliginis*	South Coronado Island	0.54 (0.5–0.6)	0.58 (0.5–0.65)	0.62 (0.55–0.7)	28 ± 3	0.53 ± 0.06	α	Negative
*C. helleri helleri*	Mainland	***	0.26	***	32 ± 2.5	0.94 ± 0.05	α, β	Positive
*C. catalinensis*	Santa Catalina Island	***	1.47 (1.4–1.5)	***	145 ± 14	1.4 ± 0.07	α, β	Negative
*C. pyrrhus muertensis*	El Muerto Island	***	1.16 (1.0–1.3)	***	165 ± 12.5	1.98 ± 0.16	α, β	Negative

* Units of enzymatic activity per milligram of venom ± standard deviation, ** fibrinolytic degradation of α, β, or γ chain, *** not determined.

**Table 2:** Neutralization capacity of commercial and experimental antivenoms in Mexico against *C. helleri caliginis* venom.

Antivenom	Batch	ED_50_ (µLAV/3LD_50_)	mgAV/mgV	LD_50_/Vial
Antivipmyn^®^	B-8K-31	40 (25.8–55.6) *	6.4	789
Faboterapico polivalente antiviperino^®^	FV044A	34.3 (27.8–40.9) *	29	875
Inoserp^®^	8805181002	92.4 (90.6–94.3) *	21.7	324

ED_50_: effective dose 50%: neutralizing potency expressed as microliters of antivenom that neutralized 3 times the median lethal dose; mg AV/mg V: milligrams of antivenom needed to neutralize 1 mg of venom; LD_50_/vial: LD_50_ neutralized per vial. * Mice presented hind limb spastic paralysis during neutralization studies.

## Data Availability

The authors confirm that the data supporting the findings of this study are available within the article and/or its Supplementary Materials, but also in the PhD thesis of Cristian Alejandro Franco Servín in the Universidad Autónoma de Aguascalientes, México.

## Supplementary Materials

The following are available online at https://www.mdpi.com/article/10.3390/toxins13080582/s1, Figure S1: One dimensional SDS-PAGE at 15% analysis of *C. helleri caliginis, C. catalinensis, C. p. muertensis, C. helleri helleri, C. viridis, C. cerberus, C. lutossus* and Crotamine positive (+) of *Crotalus molossus* under reducing conditions (A) and non-reducing conditions (B). Only *C. helleri caliginis* and *C. helleri helleri* were positive to crotamine. A total amount of 20 µg of *Crotalus* venoms were loaded and only 5 µg of crotamine +. Figure S2: Two-dimensional SDS-PAGE of *C. helleri caliginis* venom under reducing conditions. A total of 150 µg was loaded on a IPG strip 3-10 isoelectric point range and 15% of polyacrylamide. Juvenile (A) and adult venom (B). Figure S3: Mass spectrometric characterization of the *C. helleri caliginis* RP-HPLC fractions 3-8. The MSI analysis yielded nine molecular masses corresponding to the possible crotamine isoforms (A–F). Figure S4: Two-dimensional SDS-PAGE of *C. helleri caliginis* venom under reducing conditions. A total of 100 µg was loaded on a IPG strip 4-7 isoelectric point range and 15% of polyacrylamide.
