# Supplementary material for: Biological and Biochemical Characterization of Coronado Island Rattlesnake (Crotalus helleri caliginis) Venom and Antivenom Neutralization
Source: Toxins (Basel). 2021 Aug 21;13(8):582. doi: 10.3390/toxins13080582 (PMC8402616; doi:10.3390/toxins13080582)
Supplement: Supplementary file 1 [file toxins-13-00582-s001.zip › toxins-1286282.pdf]

Supplementary Materials: Biological and Biochemical Characterization of Coronado Island Rattlesnake (*Crotalus helleri caliginis*) Venom and Antivenom Neutralization

Cristian Franco-Servín, Edgar Neri-Castro, Melisa Bénard-Valle, Alejandro Alagón, Ramsés Alejandro Rosales-García, Raquel Guerrero-Alba, José Emanuel Poblano-Sánchez, Marcelo Silva-Briano, Alma Lilián Guerrero-Barrera and José Jesús Sigala-Rodríguez

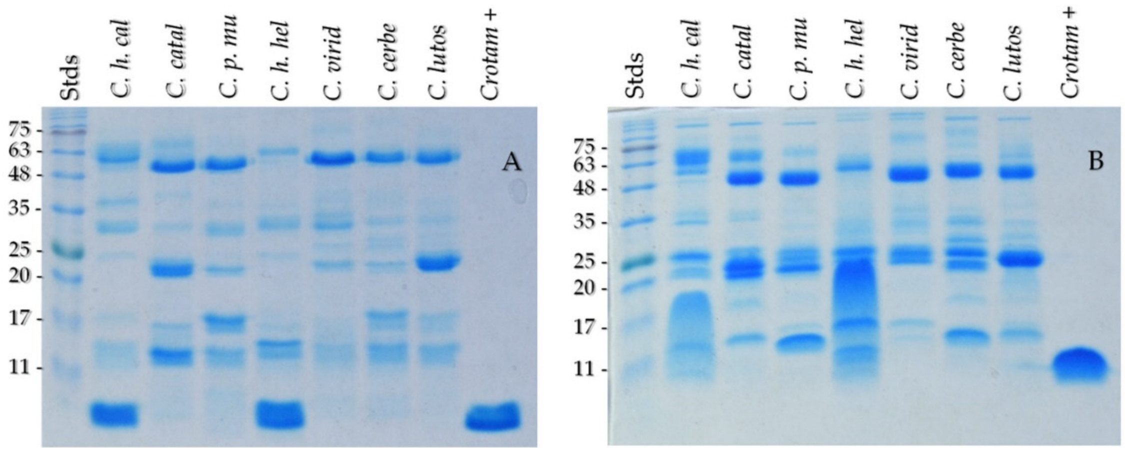

Figure S1. One dimensional SDS-PAGE at 15% analysis of *C. helleri caliginis, C. catalinensis, C. p. muertensis, C. helleri helleri, C. viridis, C. cerberus, C. lutossus* and Crotamine positive (+) of *Crotalus molossus* under reducing conditions (**A**) and non-reducing conditions (**B**). Only *C. helleri caliginis* and *C. helleri helleri* were positive to crotamine. A total amount of 20 μg of *Crotalus* venoms were loaded and only 5 μg of crotamine +.

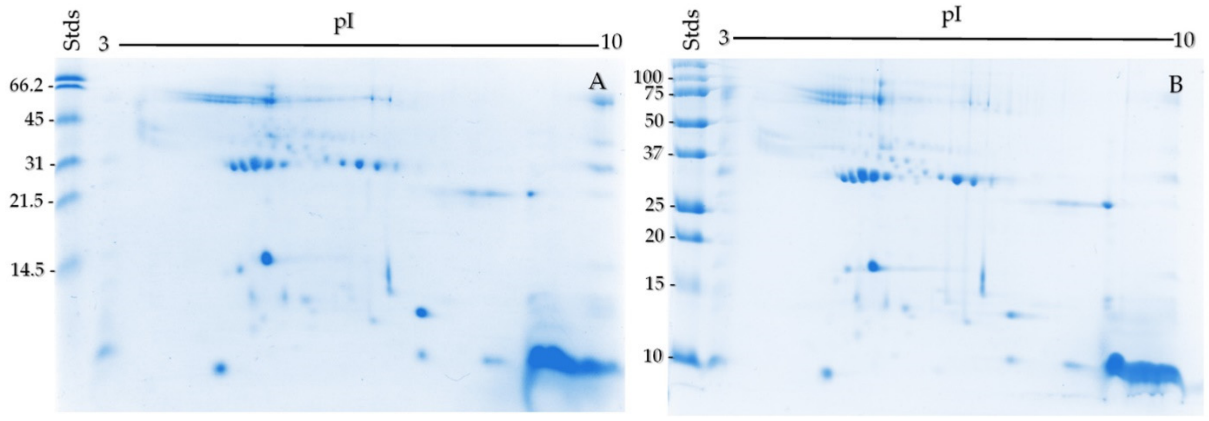

Figure S2. Two-dimensional SDS-PAGE of *C. helleri caliginis* venom under reducing conditions. A total of 150 μg was loaded on a IPG strip 3-10 isoelectric point range and 15% of polyacrylamide. Juvenile (**A**) and adult venom (**B**).

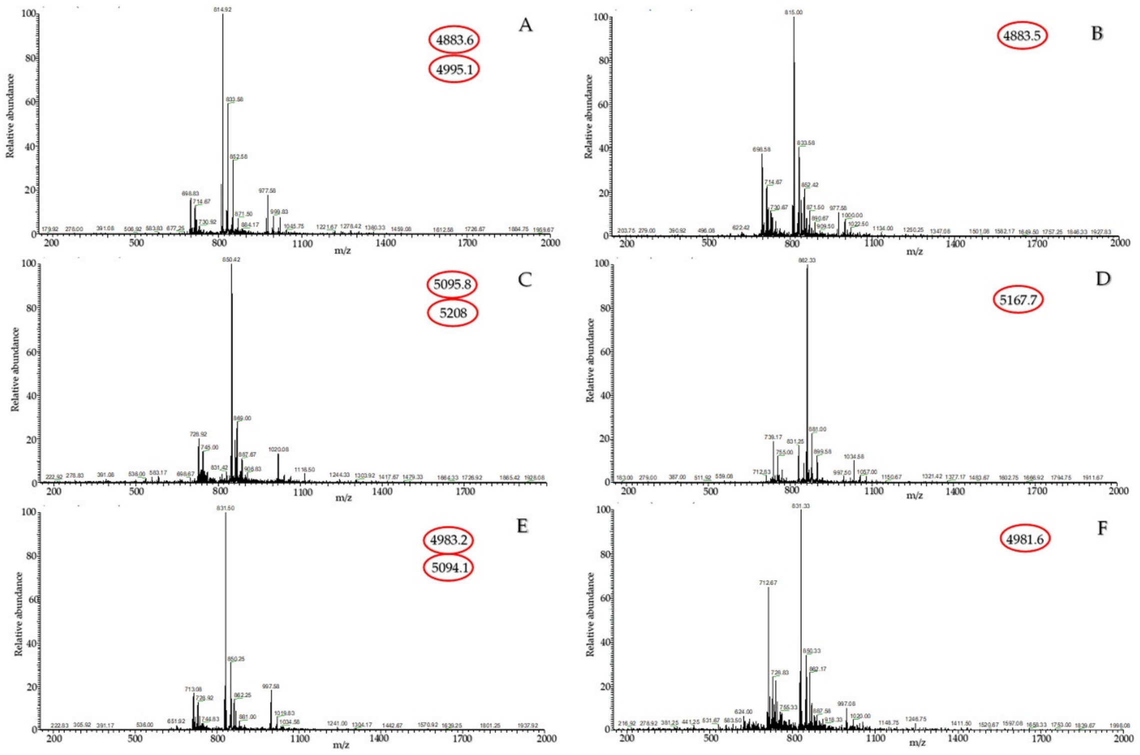

Figure S3. Mass spectrometric characterization of the *C. helleri caliginis* RP-HPLC fractions 3–8. The MSI analysis yielded nine molecular masses corresponding to the possible crotamine isoforms (**A**-**F**).

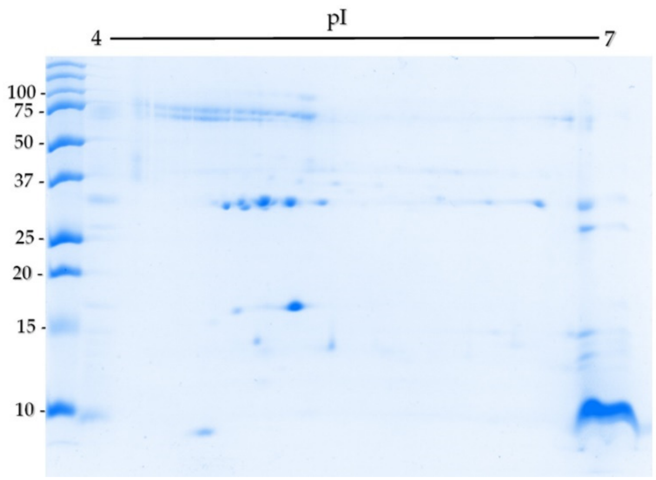

Figure S4. Two-dimensional SDS-PAGE of *C. helleri caliginis* venom under reducing conditions. A total of 100 μg was loaded on a IPG strip 4-7 isoelectric point range and 15% of polyacrylamide.
